# The complexity of hemodynamic response to the tilt test with and without nitroglycerine provocation in patients with vasovagal syncope

**DOI:** 10.1038/s41598-018-32718-2

**Published:** 2018-09-28

**Authors:** Katarzyna Buszko, Agnieszka Piątkowska, Edward Koźluk, Tomasz Fabiszak, Grzegorz Opolski

**Affiliations:** 1 Department of Theoretical Foundations of Bio-Medical Science and Medical Informatics, Collegium Medicum, Nicolaus Copernicus University, 85-067 Bydgoszcz, Poland; 2 Department and Clinic of Emergency Medicine, Wroclaw Medical University, Wroclaw, 50-556 Poland; 3 1st Chair and Department of Cardiology, Medical University of Warsaw, Warsaw, 02-091 Poland; 4 Department of Cardiology and Internal Diseases, Collegium Medicum, Nicolaus Copernicus University, 85-067 Bydgoszcz, Poland

## Abstract

The paper presents a comparison of vasovagal syndrome occurrence in a head up tilt table test between patients with a positive result of passive tilt test and those with a positive result after pharmacological provocation. The study group consisted of 80 patients: 57 patients who experienced syncope in the passive phase of the test (43 women (aged: 35.6 ± 16.2) and 14 men (aged: 41.7 ± 15.6) and 23 patients who experienced syncope after pharmacological provocation (17 women (age: 32.3 ± 12) and 6 men (age: 43 ± 15). The main investigation was based on the assessment of monitored signals complexity: heart rate, blood pressure and stroke volume. The analysis of complexity in chosen measurement phases was performed with Sample Entropy. The investigation showed that the reactions of autonomic nervous system during tilt test and before syncope are similar for positive result of passive tilt test and positive result of tilt test with provocation. The differences in supine position occurred only in analysis based on impedance measurement (SV: p = 0.01). Significant differences were denoted for all signals just before the syncope (RRI, sBP, dBP: p = 0,00001 and SV: p = 0.01). In analysis of signals complexity the significant differences occurred just before the syncope for Sample Entropy of blood pressure (SampEn (sBP): p = 0.0008, SampEn (dBP): p = 0,0001).

## Introduction

Syncope is a common cause of emergency visits and hospital admissions^1^. Each syncope episode is an indication for a more in-depth diagnostic work-up. Syncope is defined as a temporary, self-terminated loss of consciousness, usually resulting in a fall. The direct cause of syncope is temporary reversible global cerebral hypoperfusion^2^. According to the classification three types of syncope are distinguished: cardiac, orthostatic and neuro-cardiogenic^3^. Our investigation focused on the neuro-cardiogenic type which includes vasovagal syncope. This type of syncope is usually triggered by strong stress or emotions, prolonged upright tilt, especially in stuffy rooms or following vein puncture^3^. Women have higher morbidity rates of vasovagal syndrome than men^4,5^. When it does occur in younger people, the loss of consciousness might be preceded with a sensation of sudden, severe weakening, scotoma, nausea, headache, sweating or even heart palpitations. The symptoms of vasovagal syndrome in elderly people are different than in the young and rather atypical^6,7^.

The physiological mechanism of vasovagal syncope is still not fully understood. There are some theories explaining its etiology, but the principal cause remains unknown^7–10^. A typical diagnostic procedure in syncopal episodes is based on Head Up Tilt Test (HUTT) usually performed with Task Force Monitor (TFM) - a device commonly used for assessment of neuro-cardiogenic syncope^11–13^. During the test the patient lies on a table that can tilt to different angles (60 to 90 degree angle). Although this procedure has been used since 1986^14^, it has not been accepted as the gold standard diagnostic procedure for vasovagal syncope, most likely due to its limited specificity and sensitivity^15^. On the other hand, there is no alternative procedure for diagnosing syncope. Additionally, there is no single recommended protocol of HUTT. The most common are the Westminster and Italian protocols. In each of them the time in supine and tilt position as well as the angle of the tilt are precisely defined, however some clinicians perform the tilt test gauged by their own knowledge and experience.

If during a passive-standing phase (lasting 45 minutes) syncope does not occur, nitroglycerine (ntg) sublingually is administered (0.4 mg) for an additional 20-minuts tilt duration. If syncope does not occur during this time, the result of the test is considered negative. Nitroglycerin is commonly administered to increase the diagnostic yield of the HUTT. As a strong venodilators, nitrates might facilitate vasovagal reaction by enhancing venous pooling in the upright posture. Nitroglycerine given sublingual as an aerosol works approximately 1 minute, and the effect lasts for approximately 2 hours.

In the head up tilt test one creates artificial conditions that correspond to upright position lasting long enough to provoke syncope. The TFM allows recording the patient’s electrocardiogram (ECG), impedance cardiogram (ICG), and blood pressure (BP). TFM implements many algorithms for data processing in real time: HRV parameters, cardiac parameters, baroreceptors activity, etc. When syncope occurs, the analysis of baseline signals (ECG, blood pressure) allows the clinician to classify the patient response to orthostatic stress according to the VASIS classification (Vasovagal Syncope International Study)^2^. Extensive attempts have been made to describe the changes in monitored parameters seen at various time intervals after the tilt and before the occurrence of syncope. Usually the investigations focused on identification of differences in heart rhythm, blood pressure and impedance between patients with positive and negative HUTT test results. Nonetheless, it would be more diagnostically useful to be able to predict occurrence of syncope based solely on the reaction to orthostatic stress, without the necessity of a long-term tilt. For this reason, we focused our investigation on this aspect of syncope diagnosis. In the standard linear analysis the comparison of those two groups HUTT(+) vs. HUTT(−) shows no significant difference^16^. Therefore at present many investigators focus on applications of nonlinear analysis of heart rate regulation^17,18^. It is known that a variety of factors influence the heart activity, hence the classical linear analysis in time-domain as well as frequency-domain methods are inadequate to explain the complexity of heart rhythm^17^. By contrast, the nonlinear methods give us a chance for a better understanding of biomedical time series. The most popular nonlinear methods are: Poincare plots, fractal methods, symbolic dynamics measures, heart rate asymmetry and entropy^16,17,19,20^.

In this paper we focus on the nonlinear methods with special attention paid to entropy measurement. We present here the application of one kind of entropy: Sample Entropy (SampEn) to assess tilt table test results in patients suspected of having vasovagal syndrome. The paper is arranged as follows: section I describes vasovagal syndrome, section II presents Sample Entropy, section III characterizes materials and methods, section IV shows the results of research and section V contains discussion on the obtained results.

## Vasovagal Syndrome

A syncope can be specified as a temporary self-terminated loss of consciousness. In most cases it results in a fall. The short-term reversible general brain ischaemia is assumed to be a direct cause of syncope. Due to this reason, one must differentiate syncope from cases when loss of consciousness occurs due to causes not related with ischeamia, e.g. epilepsy, hyperventilation with hypocapnia, hypoglycaemia, poisoning, psychogenic reaction that are often mistakenly diagnosed as syncope. In Europe, cases of syncope account for about 1% of admissions to the Emergency Department^21–27^. About 40% of this group is hospitalized^23^. In about 47% of women and 31% of men, the first episode of syncope occurs in the age between 10 and 30 years^5^, and most of them have reflex syncope. The second peak of incidence of syncope is observed in patients over 65 years old^5^. About 1/3 of patients suffer from more than one fainting episode within 3 years. The majority of patients have prodromal symptoms of varying duration, including dizziness, nausea, perspiration, weakness, visual disturbances occurring prior to syncope. Nonetheless, loss of consciousness often arrives without warning. Three types of syncope can be distinguished in terms of its ethology: neurocardiogenic, orthostatic and cardiogenic. Neurocardiogenic (reflex) syncope can be further classified according to the triggering stimulus to vasovagal (associated with orthostatic stress or emotion), situational (after micturition, sneezing, physical activity, early autonomic instability), carotid sinus hypersensitivity and atypical (reflex syncope occurs after specific, sometimes unclear trigger stimuli or even without any)^2^. The autonomic malfunction causing reflex syncope is either a vasodepressive response (loss of sympathetic vasoconstrictive tone with lowering of blood pressure) or cardio-depressive response (active parasympathetic stimulation leading to bradycardia or asystole) resulting in impaired cerebral perfusion^2^.

Situations that can provoke fainting vary considerably between and within individuals. In most cases, the type of reaction does not depend on the type of stimulus. Moreover, an individual patient may demonstrate different types of responses on different occasions. The pathophysiology of neurocardiogenic syncope is heterogeneous. The experience gained so far and assessment of the results of tilt test show that a short pause in cerebral perfusion (lasting 6–8 s) or a decrease in systolic blood pressure (sBP) down to 60 mmHg or less is associated with syncope^28^. The sBP depends on cardiac output (CO) and total peripheral resistance (TPR). A drop in any of these parameters can cause syncope. Usually, both of these BP determinants decrease, but the role of each individual mechanism is significantly different.

There are two physiological theories attempting to explain the occurrence of vasovagal syncope. According to the central theory by van Lieshout and al., the reflex is triggered by activation of the cortical-subcortical centers, participated by necrohormones and neurotransmitters which lead to the bradycardia-hypotension reflex due to factors such as pain, fear or emotions^29^. The peripheral theory by Oberg and Thoren assumes that the reflex is triggered by stimulation of the cardiac mechanoreceptors in the left ventricle, cardiac atria and aortic arch as well as peripheral vascular chemoreceptors due to prolonged maintenance of upright body position^30^. Both theories agree that the cause of syncope is central hypovolemia resulting from deposition of blood in lower extremities and skeletal muscles, leading to a drop in blood pressure, bradycardia, and a drop in skeletal muscle tension. These two and other less popular theories show that the pathophysiological mechanism of vasovagal syndrome is still not fully determined. Therefore applications of new, more advanced methods for syncope analysis are continuously tested in search for better understanding of its mechanisms. To this end we propose an analysis based on entropy measures and we have chosen sample entropy as an example.

## Sample Entropy

Entropy is a concept introduced by Rudolf Clausius in thermodynamics as a measure of system uncertainty^31^. The idea of entropy was implemented to information theory and applied to describe loss of data in information transmission systems. The mathematical formula was proposed by Shannon and is known as Shannon Entropy^32^. As a consequence of the development of information theory and time series analysis, many formulas and methods of entropy calculation appeared in a short period of time. Despite that, Shannon Entropy is still a widely known formula of entropy.

Entropy started to be implemented also in biomedical signals analysis as a parameter of their complexity. Entropy for heart rhythm analysis was first proposed by Pincus and it was named Approximate Entropy (ApEn)^33^. The idea of ApEn was based on measuring the likelihood that the similar sequences of points in time series remain similar for increment sequences^33^. The estimation of ApEn was used in many cardiovascular studies^34–37^, but it is known that, it has some limitations. ApEn counts self-matches generating a bias towards regularity^38,39^. This results in dependency on the time series length and the loss of relative consistency. The mentioned limitations inspired Moormann and Richmann to develop a calculation of entropy. They proposed the idea of Sample Entropy (SampEn)^38^. The algorithm for SampEn calculation is well known. Here we briefly describe its mathematical basis^17,38^.

Let us take the time series of length N, mark the length of the basic sequence as m and the similarity criterion as r. For the time series

21 $${\{{x}_{i}\}}_{i=1}^{N}=[{x}_{1},\,{x}_{2},\,{x}_{3},\,\ldots .\,{x}_{N}]$$

we define a vector

22 $${p}_{m}(i)=({x}_{i},\,\ldots ,\,{x}_{i+m-1})\,\,\,\,\,\,i\le N-m+1$$

And a set of vectors is formed:

23 $${P}_{m}=\{{p}_{m}(1),\,{p}_{m}(2),\,\ldots ,\,{p}_{m}(N-m+1)\}$$

Let us define the distance between two vectors ${p}_{m}(i),\,{p}_{m}(j)$ as:

24 $${d}_{i,j}^{m}=\,max|{x}_{i+k}-{x}_{j+k}|\,\,\,\,\,\,\,0\le k\le m-1$$

The vectors ${p}_{m}(i),\,{p}_{m}(\,j)$ are similar if they fulfill the similarity criterion:

25 $${d}_{i,j}^{m}\le r\,\,\,\,\,{\rm{w}}{\rm{h}}{\rm{e}}{\rm{r}}{\rm{e}}\,\,\,\,1\le i,\,\,j\le N-m\,\,\,\,{\rm{w}}{\rm{h}}{\rm{e}}{\rm{r}}{\rm{e}}\,\,\,i\ne j$$

Let us define ${B}_{i}^{m}(r)$ as the number of $\,{p}_{m}(\,j)$ vectors that are similar to vector ***p***_***m***_(***i***) (fulfilling the criterion (2.5) and similarly ${A}_{i}^{m}(r)$ for $m+1$ that fulfill the criterion $({d}_{i,j}^{m+1}\le r)$. Sample Entropy (***SampEn***(***m***, ***r***, ***N***)) is defined by:

26 $$SampEn(m,\,r,\,N)=-\,{\rm{l}}{\rm{n}}(\frac{\sum _{i=1}^{N-m}{A}_{i}^{m}}{\sum _{i=1}^{N-m}{B}_{i}^{m}})$$

The parameters $m$ and $r$ are usually chosen arbitrarily. In cardiovascular physiology^35–45^ the embedding dimension m is usually set at 2 and following Pincus, the threshold is set to r = 0.2 × SD, where *SD* is the standard deviation of ***x***_***i***_^33^. The SampEn (m, r, N) statistics does not count self-matches. It has better theoretical agreement than ApEn, especially for random numbers with known probabilistic properties and short data. It also provides better relative consistency. Both ApEn and SampEn have extensive applications in cardiovascular physiology^38–43^. In our previous paper^46^ we presented an example of application of both ApEn (m, r, N) and SampEn (m, r, N) statistics for analysis of physiological signals. In our current investigation we focus on the assessment of signals complexity in short time intervals and for this reason the choice of SampEn statistic is necessary. The choice of SampEn in assessment of VVS analysis was inspired by the interesting results obtained by others researchers^35–45^.

## Materials and Methods

### Study group

It is a single-centre, retrospective analysis performed on a group of 230 patients diagnosed in Cardiology Outpatient Clinic at the 1st Chair and Department of Cardiology, Medical University of Warsaw from 2005 to 2013. The collected database was used to search for the patients with vasovagal syncope. The diagnosis of VVS was based on Westminster Protocol^2^ and classified according to the VASIS classification^47^. The study group consisted of 80 patients under investigation for vasovagal syncope with the tilt test due to recurrent syncope. The study was conducted only on patients suffering from neuro-cardiogenic syncope. Patients with heart and brain diseases were excluded. Based on the result of passive HUTT (positive vs. negative) the study group was divided into two separated sub-groups signified respectively as HUTT(+) and HUTT(−). The HUTT(+) sub-group consisted of 57 patients who experienced syncope in the passive phase of the test. There were 43 women (aged: 35.6 ± 16.2) and 14 men (aged: 41.7 ± 15.6). Among the female participants the youngest was 18 years old and the oldest was 66. Among the male participants the youngest was also 18 years old and the oldest was 59. The HUTT(−) group consisted of 23 patients who passed the passive phase of the test uneventfully, but experienced syncope after pharmacological provocation with nitroglycerine. In this group there were 17 women (age: 32.3 ± 12) and 6 men (age: 43 ± 15). The youngest female was 20 years old and the youngest man was 22 years old, while the oldest male and female were 56 and 62 years old, respectively.

The study was conducted in accordance with the principles included in the Declaration of Helsinki. All the participants of the study provided a written conscious consent prior to any specific procedures of the study and agreed to use clinical data for scientific purposes. The study was approved by The Ethics Committee of Medical University of Warsaw, Poland (approval number AKBE/51/2018).

### Measurements

The Task Force Monitor system device (CNSystem, Graz, Austria) was used to perform the HUTT in each case. The measurements were conducted in the morning in a quiet room with dimmed lighting. The patients were fasting prior to the test. There was used protocol based on the modified Westminster protocol^2^, i.e. patients remained in the supine position for 20 minutes and afterwards they were tilted to 60 degrees in 5 seconds. The passive upright tilt was terminated upon syncope occurrence, otherwise its duration was 45 minutes. The test was considered positive if syncope or pre-syncope was accompanied by bradycardia or hypotension. In case of asymptomatic hypotension or bradycardia the test was regarded as suggestive of neurocardiogenic reflex. In absence of syncope, the test was prolonged by additional 20 minutes and pharmacological provocation with 0.4 mg nitroglycerine (ntg) administered sublingually was carried out^48^. There were three cases when syncope did not occur after 45 minutes, but a significant drop in the sBP at the end of the passive upright tilt to values below 100 mmHg was recorded, marking the onset of the neuro-cardiogenic reaction. In those cases it was decided to prolong by another 15 minutes the passive tilt phase. During the added time each of the three patients eventually experienced syncope. In that cases the low blood pressure, was a contraindication to giving the nitroglycerine. A full syncope or a pre-syncope condition achieved without pharmacological provocation (HUTT(+) indicated the end point of the passive test. The minimum and maximum time from the tilt to syncope was 3 and 52 minutes, respectively. After the tilt, the syncope was noted on average after 20 minutes of time. In the prolonged test with pharmacological provocation (ntg) the minimum time from the provocation to syncope was about 2 minutes, while the maximum time was 6.8 minutes. The average time from administration of nitroglycerine to syncope was 3.8 minutes. Based on the HUTT results, the observed changes in heart rhythm and blood pressure for each patient during the neuro-cardiogenic reaction were allocated according to the VASIS classification^47^.

### Data Analysis and statistical methods

We analyzed three basic physiological biosignals recorded with the TFM during HUT test. These were: high resolution ECG (Electrocardiography) signal (2-channels with a sampling frequency of 1000 Hz), systolic and diastolic blood pressure (sBP, dBP) measured continuously and ICG (impedance cardiography). We based our analysis on the RR-intervals (RRI) extracted from the ECG recordings. The TFM system determined the time interval from opening to closing of the aortic valve (LVET) and stroke volume (SV) from the impedance curve. All the signals were recorded with beat-to-beat time intervals and the measuring time points were indicated by the cardiac rhythm. The clinician carefully checked all the signals. Among all the data obtained during the measurement, the abnormal results did not exceed 5% of. The data have been not detrendend.

**Figure 1:**
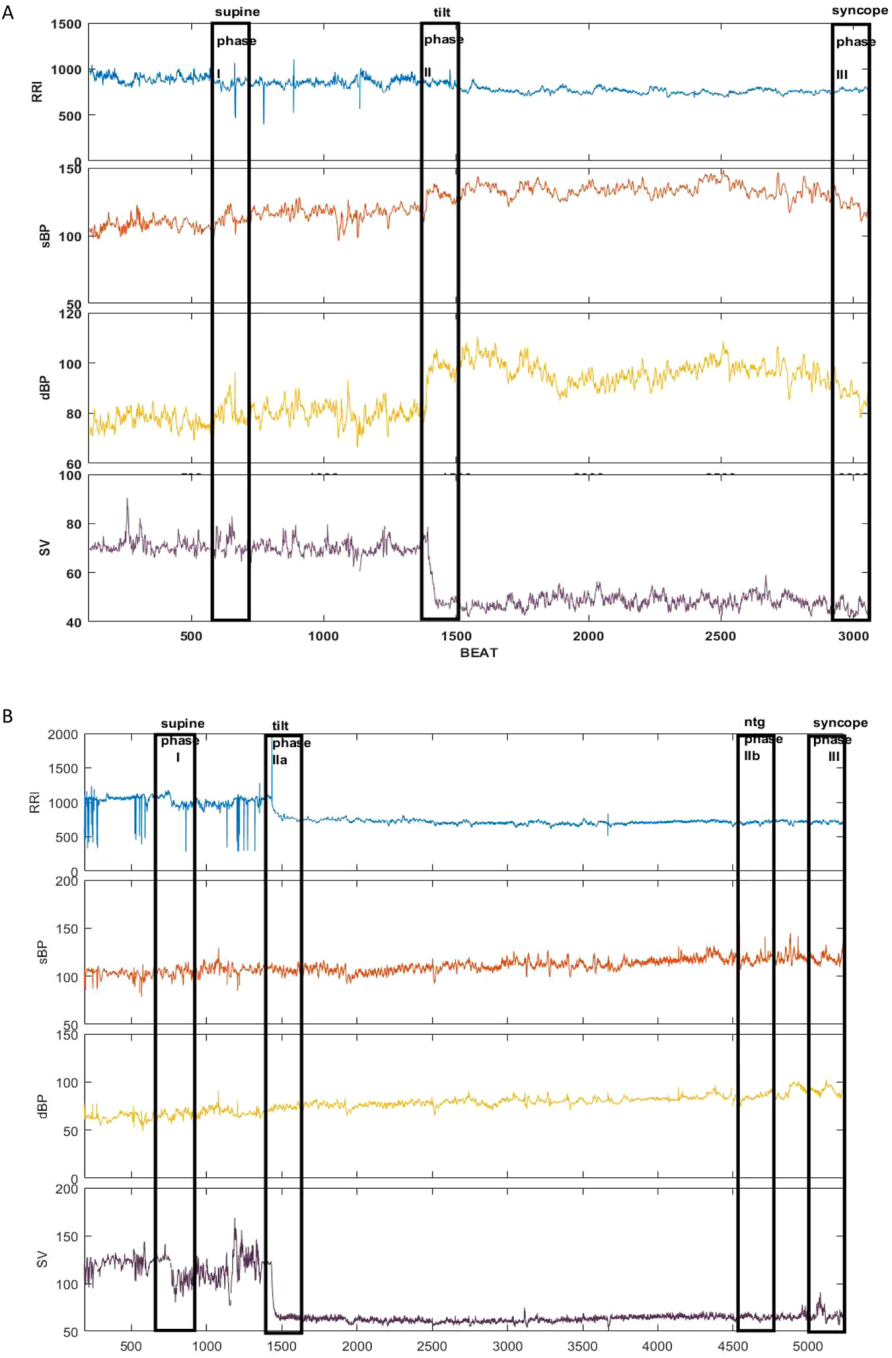
The recorded parameters (RRI, sBP, dBP, SV) and phases of measurements used for calculations for the study subgroups: (**a**) HUTT(+) (**b**) HUTT(−).

As mentioned previously, our attention was focused on the basic parameters: RRI, sBP, dBP, SV. Figure 1 presents an example of parameters recorded for single patients from the HUTT(+) (Fig. 1a) and HUTT(−) (Fig. 1b) group. The stages of the positive HUTT are marked as follows: I-supine position, IIa-tilt, III-syncope. For the negative HUTT the following coding have been used: I-supine position, IIa-tilt, IIb-ntg provocation, III-syncope. From each stage for the HUTT 250-beats intervals of data were selected, forming 250-beats registration windows. The windows are marked in Fig. 1 as solid-line boxes.

For each group the values of RRI, sBP, dBP and SV were compared between the registration windows. Afterwards, we determined SampEn of the parameters for each window. During the next step, the entropies between the windows were compared. We also determined SampEn of the shuffled data in each window and we compared them with entropies determined for real data.

The last stage of the analysis is focused on each window separately. We determined the entropies in a sliding window of 100 beats for each of the windows. We were using sliding window forwarding with 1 beat step. The sliding window idea can be described as follows:

For *N, n, i, p, k, j*, ∈ ${\mathbb{N}}$ let us define a time series of length *N* as a sequence:

3.1 $$S={\{{S}_{n}\}}_{n=1}^{N},$$

where $n\,\epsilon \,\{1,\,..\,N\},\,{S}_{n}\in {\mathbb{R}}$ – is ***n***-th sample of the chosen biosignal (RRI, sBP, dBP and SV).

Let us take a subset $W\subseteq S$ of p subsequent points of the *S* series, where 1 ≤ *p* ≤ *N* and denote its elements as follows:

3.2 $$W={\{{w}_{j}\}}_{j=1}^{p}$$

For 1 ≤ *k* ≤ *p* let us define *X* as a family of subsets of *k* elements of set *W*:

3.3 $$X={\{{X}_{i}\}}_{i=1}^{p-k+1}$$

where $$W\supseteq {X}_{i}={\{{w}_{j}\}}_{j=i}^{k+i-1}$$ for $$i=1,\,\ldots ,\,p-k+1$$

Now let us define a function *f* as follows:

3.4 $$f:\{1,\,\mathrm{..},\,p-k+1\}\to {\mathbb{R}},\,f(i)=SampEnf(m,\,r,\,k,\,{X}_{i}),$$

where $$SampEnf:{\mathbb{N}}\times {\mathbb{R}}\times {\mathbb{N}}\times X\to {\mathbb{R}}\,$$ and

3.5 $$SampEnf\,(m,\,r,\,k,\,{X}_{i})=SampEn\,(m,\,r,\,k)$$

is calculated for a given sequence *X*_*i*_ of *k-*elements according to the formula (2.6).

The function (3.4) defines the sliding window. The values of *f* are the subsequent values of the entropy calculated “beat-by-beat” in the *p*-beats windows using *k-*elements sliding window. In our investigations we applied *p* = 250, *k* = 100, *m* = 2, *r* = 0.2 × *SD*, where *SD* is the standard deviation of ${X}_{i}$. The obtained vector of entropies (for each signal) shows changes of the signal complexity for each analyzed stage of the HUTT.

We also compared the value of: RRI, sBP, dBP, SV, SampEn(RRI), SampEn(sBP), SampEn(dBP), SampEn(SV) between the HUTT(+) vs. HUTT(−) subgroups in the analyzed phases of the test. Similar calculations of entropies were performed for the shuffled data (SampEn(RRIsh), SampEn(sBPsh), SampEn(dBPsh), SampEn(SVsh) and then the comparisons between the entropies of real and shuffled data were investigated. We used nonparametric tests for statistical analysis as the statistical conditions for implementing parametric methods (normal distribution, variance equality) were not met. We compared the parameters with the Friedman test and the post-hoc multicomparison test. The value of α = 0.05 was considered significant. The algorithm from Physionet^49^ was used to determine the Sample Entropy. We did the calculations with the Matlab R2017b system.

## Results

### Parameters’ comparison for HUTT selected stages between HUTT(+) and HUTT(−) groups

**Figure 2:**
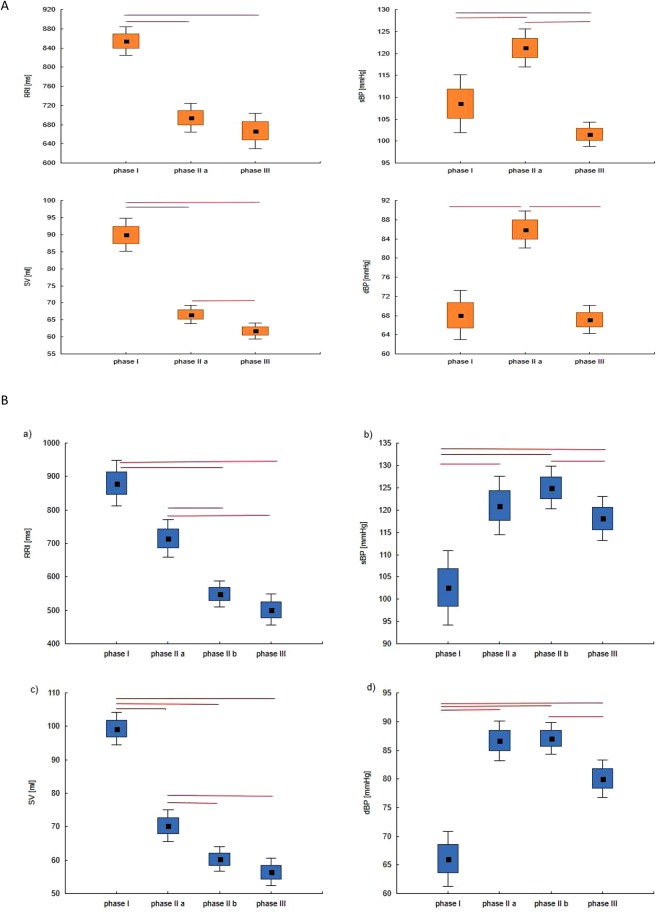
The box-plots (mean ± SEM and mean ± 1.96·SEM) and Friedmann test with multicomparison post-hoc test in tilt test for group: (**A**) HUTT(+) in phase I, II and III, (**B**) HUTT(−) in phase I, IIa, IIb, III.

In Section 3.3 we indicated the phases of the tilt test applied for the HUTT(+) and HUTT(−) subgroup (Fig. 1). Figure 2(A,B) shows box plots for analyzed parameters (RRI, sBP, dBP, SV) in the described phases for the HUTT (+) group (Fig. 2A) and for the HUTT (−) group (Fig. 2B). We performed comparisons of parameters with the Friedman test and multicompare post-hoc test. Statistically significant differences between the phases of HUTT are represented by the lines linking the boxes. The boxes present mean and SEM and the whiskers indicate the confidence interval.

**Figure 3:**
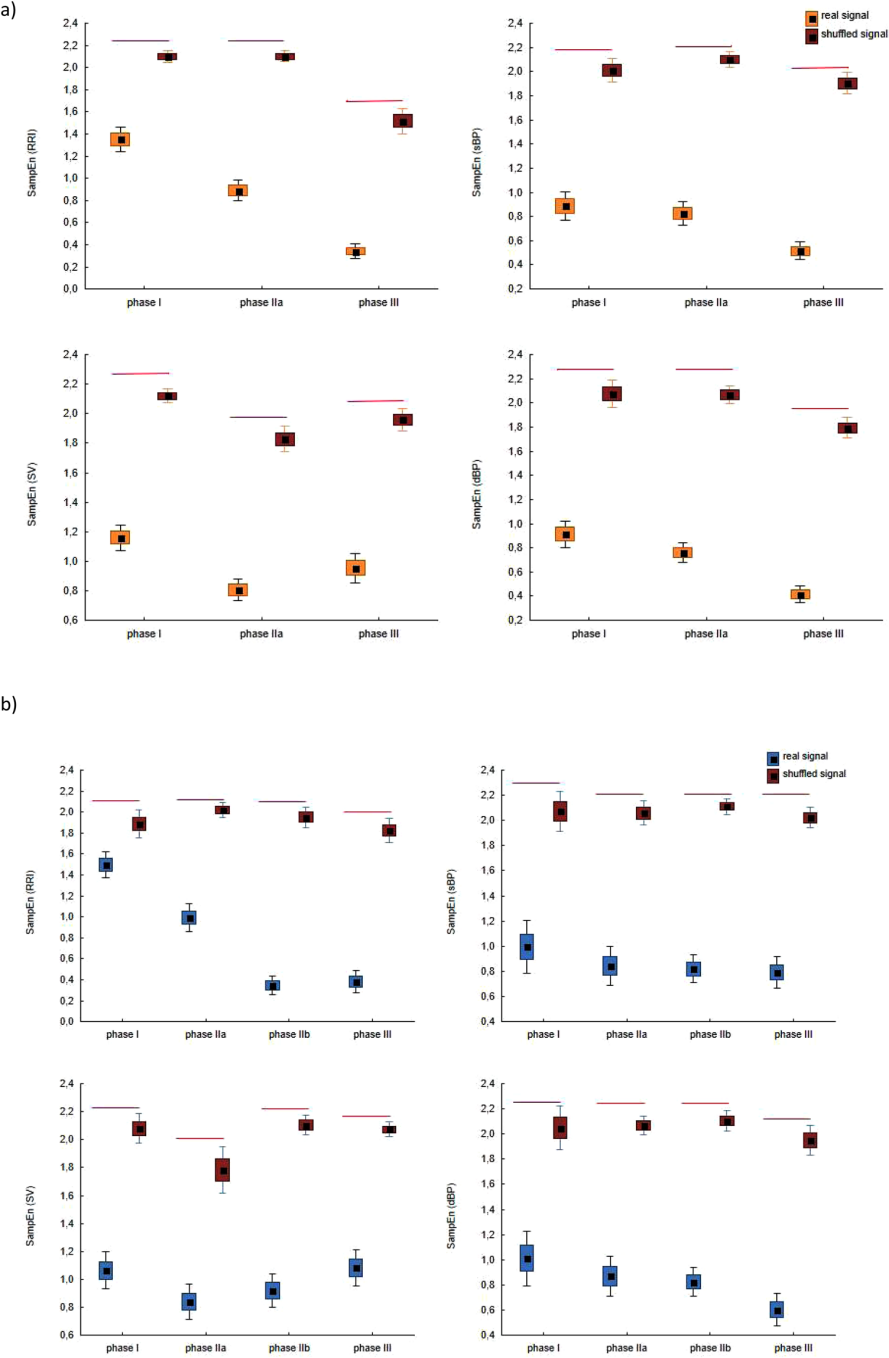
The box-plots (mean ± SEM and mean ± 1.96·SEM) and Mann-Whitney test for comparisons of SampEn (RRI), SampEn (sBP), SampEn (dBP), SampEn (SV) calculated for real signal and shuffled signal in successive HUTT phases for: (**a**) HUTT(+), (**b**) HUTT(−).

We calculated Sample Entropy in each phase for the shuffled data of RRI, sBP, dBP and SV. Figure 3 presents box plots for real and shuffled data in each HUTT phase for: SampEn (RRI)-(a), SampEn (sBP)-(b), SampEn(dBP)-(c), SampEn (SV)-(d) for group HUTT(+) (part A) and for group HUTT(−)(part (B).

**Figure 4:**
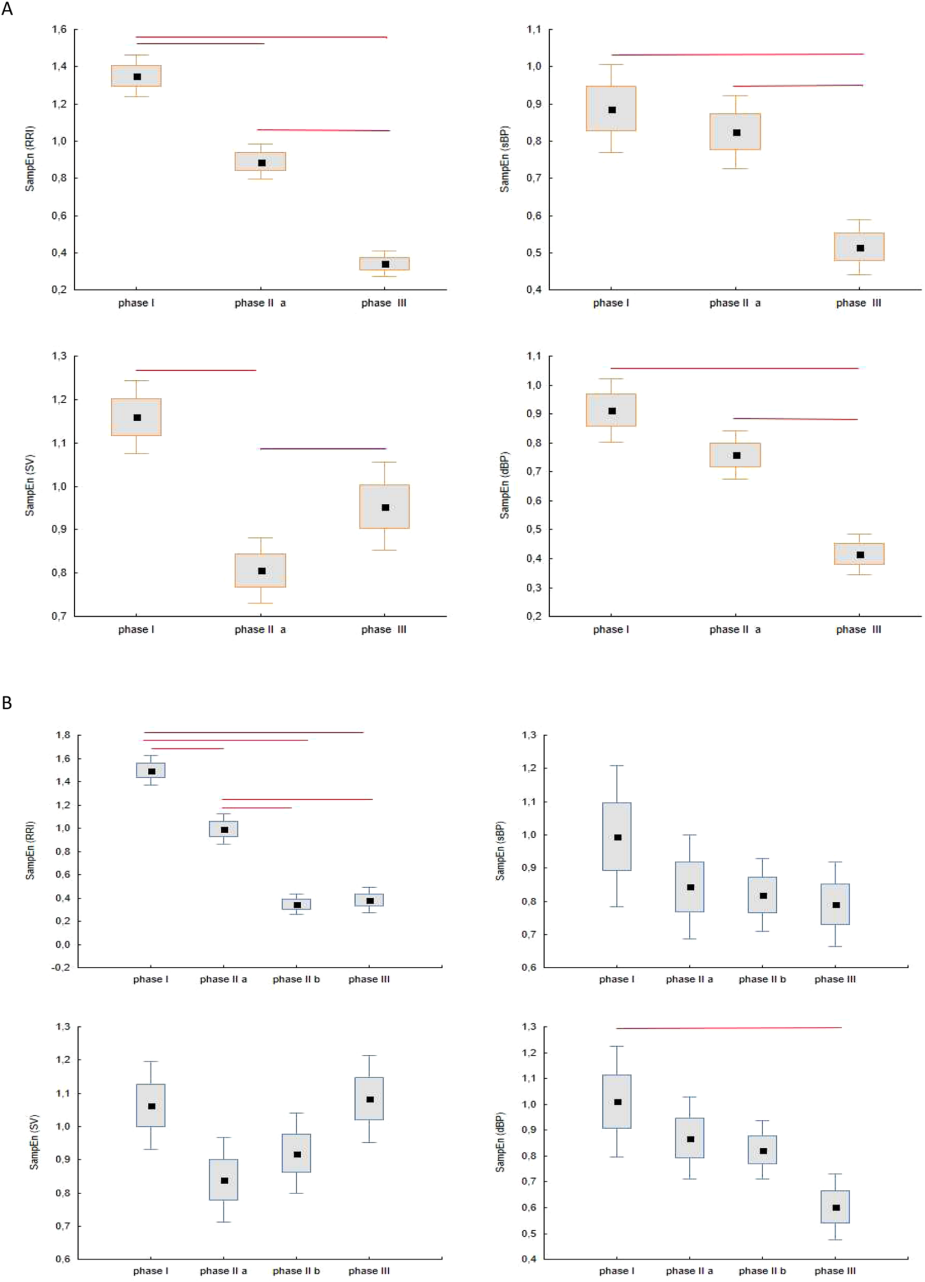
The box-plots (mean ± SEM and mean ± 1.96·SEM) and Friedmann test with multicomparison post-hoc test for SampEn (RRI), SampEn (sBP), SampEn (dBP), SampEn (SV) for the study groups: (**A**) HUTT(+) (**B**) HUTT(−).

Figure 4(A,B) presents box plots for comparison of SampEn calculated for RRI, sBP, dBP and SV in phase I, II, III for the HUTT(+) group and in phase I, IIa, IIb, III for the HUTT(−) group. In our calculations we assumed ***r*** = **0.2**, ***m*** = **2**, ***N*** = **250**, as it can be found in the literature and used by other researchers in RRI analysis^33,38^.

Table 1 shows mean values, standard deviation of the parameters and entropies in all HUTT phases for both groups and for the entropies calculated for shuffled data (RRIsh, sBPsh, dBPsh, SVsh).

**Table 1:** Descriptive statistics of HUTT(+) and HUTT(−) groups in phases I, IIa, IIb and III (mean ± sd).

Parameter	HUTT (+)			HUTT(−)			
	I	IIa	III	I	IIa	IIb	III
RRI (ms)	854.2 ± 114.7	694.7 ± 113.7	668.6 ± 143.2	880 ± 165.5	715.3 ± 135.6	549.3 ± 96.4	501.8 ± 112.8
sBP (mmHg)	108.6 ± 25.4	121.2 ± 16.8	101.6 ± 10.6	102.6 ± 20.4	120.9 ± 16	125 ± 11.6	118.1 ± 12
dBP (mmHg)	68.0 ± 19.7	85.9 ± 14.9	67.2 ± 11.2	66.1 ± 16.8	86.7 ± 12.2	87.1 ± 9.7	80 ± 11.5
SV (mL)	89.9 ± 18.7	66.6 ± 10.3	61.8 ± 9.1	99.3 ± 12	70.2 ± 11.6	60.3 ± 8.8	56.5 ± 10
SampEn (RRI)	1.35 ± 0.3	0.88 ± 0.3	0.34 ± 0.2	1.5 ± 0.3	1.0 ± 0.3	0.34 ± 0.2	0.38 ± 0.24
SampEn (sBP)	0.88 ± 0.4	0.82 ± 0.3	0.51 ± 0.3	1.0 ± 0.5	0.84 ± 0.36	0.8 ± 0.2	0.8 ± 0.3
SampEn (dBP)	0.91 ± 0.4	0.76 ± 0.3	0.41 ± 0.3	1.0 ± 0.5	0.86 ± 0.36	0.8 ± 0.26	0.6 ± 0.3
SampEn (SV)	1.16 ± 0.3	0.8 ±± 0.3	0.95 ± 0.4	1.06 ± 0.3	0.84 ± 0.3	0.92 ± 0.3	1.08 ± 0.3
SampEn (RRIsh)	2.1 ± 0.2	2.1 ± 0.2	1.5 ± 0.4	1.9 ± 0.3	2.0 ± 0.1	1.9 ± 0.2	1.8 ± 0.3
SampEn (sBPsh)	2.0 ± 0.4	2.1 ± 0.2	1.9 ± 0.3	2.0 ± 0.4	2.0 ± 0.2	2.1 ± 0.1	2.0 ± 0.2
SampEn (dBPsh)	2.1 ± 0.4	2.1 ± 0.3	1.8 ± 0.3	2.0 ± 0.4	2.1 ± 0.2	2.1 ± 0.2	1.9 ± 0.3
SampEn (SVsh)	2.1 ± 0.2	1.8 ± 0.3	1.9 ± 0.3	2.0 ± 0.2	1.8 ± 0.4	2.1 ± 0.2	2.0 ± 0.1

### Comparison of HUTT(+) and HUTT(−) groups for selected HUTT stages

**Figure 5:**
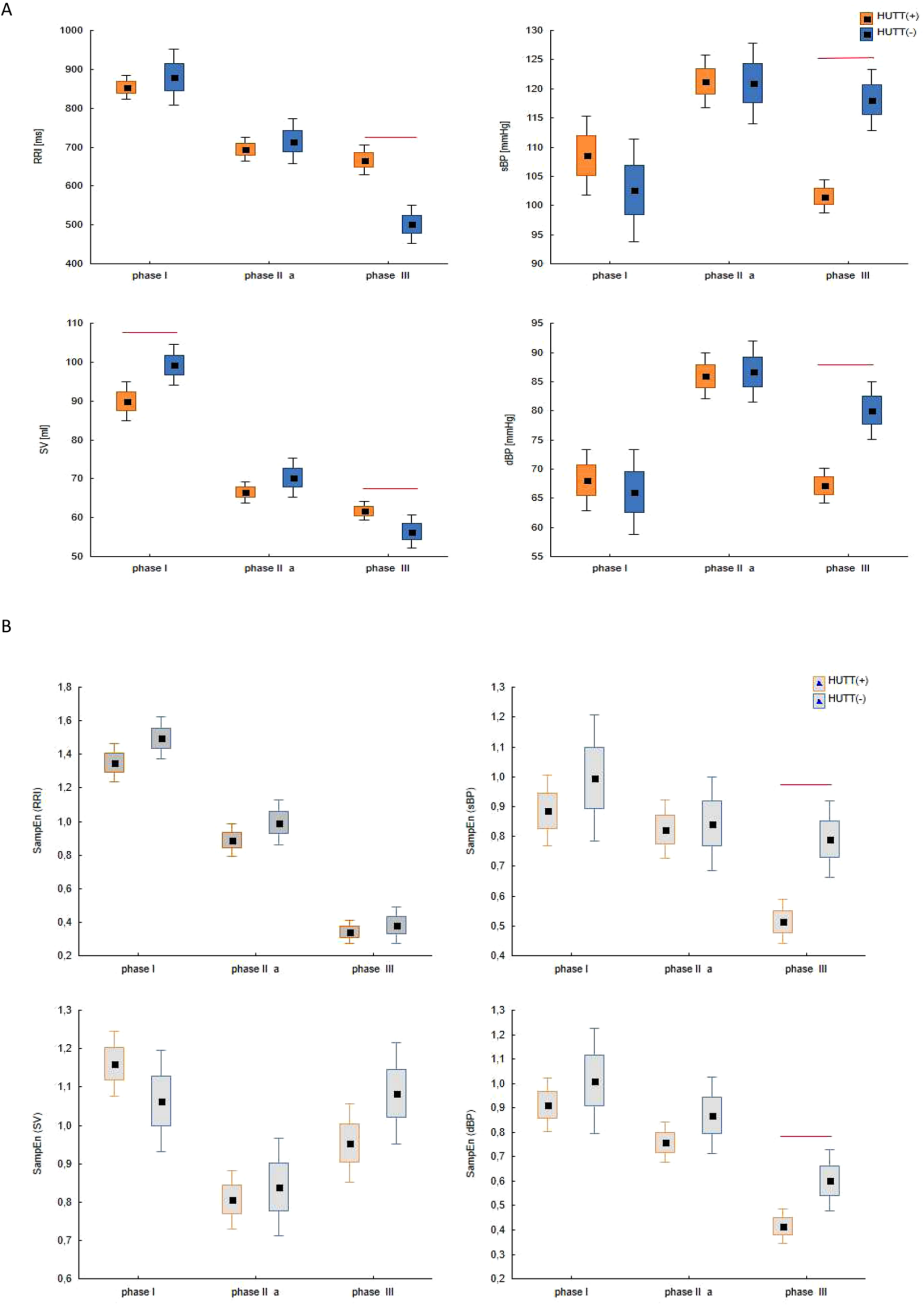
The box-plots (mean ± SEM and mean ± 1.96·SEM) and Mann-Whitney test for comparisons of the study groups (HUTT(+) vs. HUTT(−) in successive HUTT phases for: (**A**) RRI, sBP, dBP, SV, (**B**) SampEn (RRI), SampEn (sBP), SampEn (dBP), SampEn (SV).

The tilt test was performed for each patient in accordance with the Westminster protocol. The difference in the tilt procedure between patients from the HUTT(+) vs. HUTT(−) group manifested in the duration of the test and the use of nitroglycerin provocation. Figure 5A presents comparisons of box plots for the analyzed groups and parameters (5(a)-RRI, 5(b)-sBP, 5(c)-dBP, 5(d)- SV) in each HUTT phase. Figure 5B presents box plots for both groups in each HUTT phase for: SampEn (RRI)-(a), SampEn (sBP)-(b), SampEn(dBP)-(c), SampEn (SV)-(d). We used the Mann-Whitney test to compare the parameters. Statistically significant differences between the groups are represented by the red lines linking the boxes.

### Sample Entropy in sliding windows

The major changes in RRI, sBP, dBP and SV occurred in phase III that ended with the occurrence of syncope. As it was mentioned before, a drop in sBP and dBP are the main indicators of syncope.

**Figure 6:**
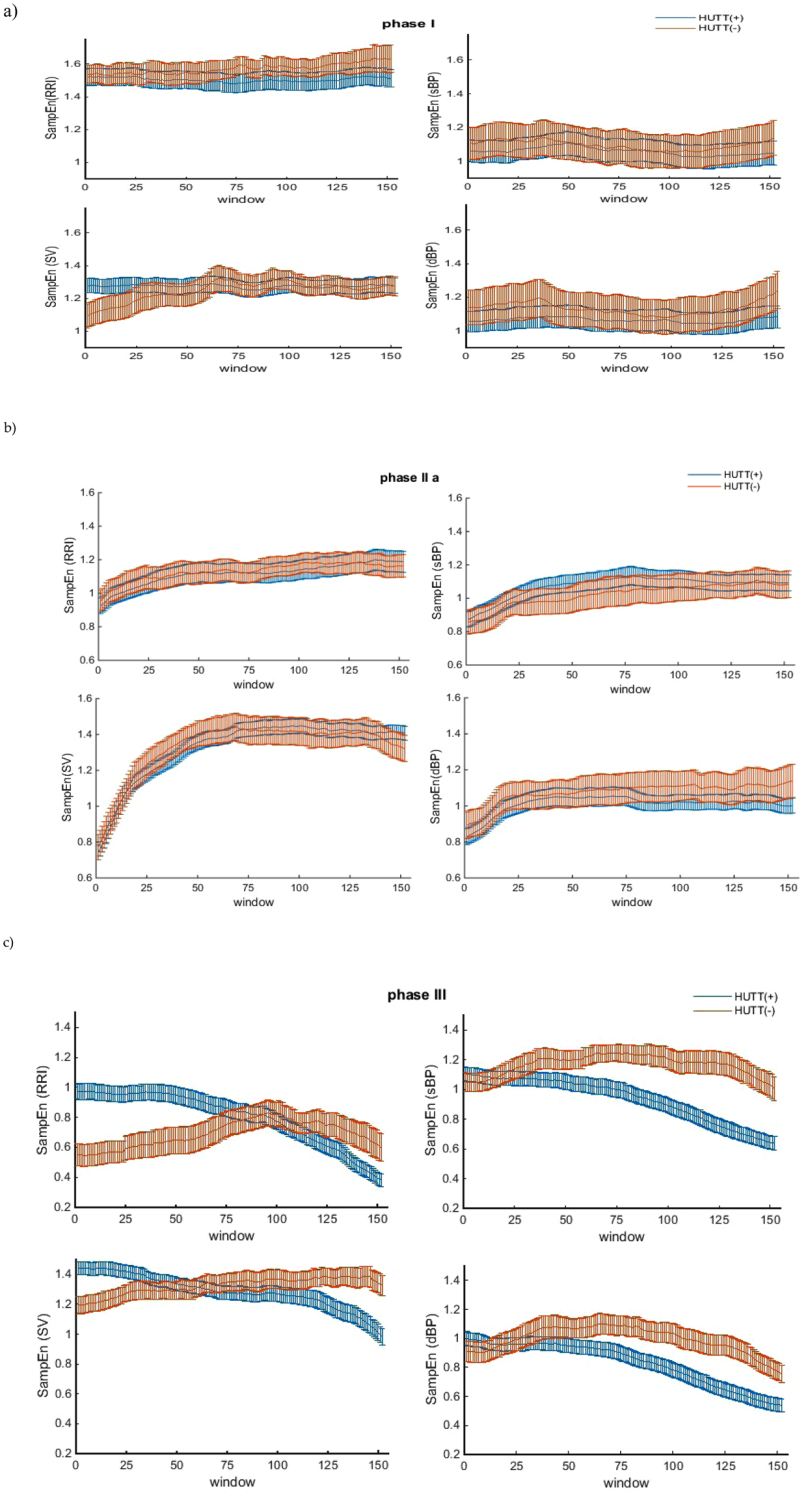
Comparisons of average SampEn with standard error (SEM) in sliding windows between the HUTT(+) and HUTT(−) groups in successive HUTT phases: (**a**) I, (**b**) IIa (**c**) III.

We also investigated changes in the dynamics of entropy in a sliding window of 100 beats, We determined SampEn for the previously mentioned parameters (RRI, sBP, dBP, SV). In all cases, SampEn was set for *r* = 0.2 × *SD*, *m* = 2, *N* = 100. The sliding window was advancing by 1 beat step each time. Figure 6(a–c) presents plots for SampEn (RRI), SampEn (sBP), SampEn (dBP) and SampEn (SV) in a sliding window for the HUTT(+) and HUTT(−) groups together, thus allowing comparison of the dynamics of SampEn changes between the groups in each phase.

The discussion of the study results is presented in Section 5.

## Discussion

The physiological mechanism of vasovagal syndrome is complex and despite many investigations it has not been fully elucidated. Its full explanation would improve the diagnostics of vasovagal syndrome and add to more efficient help for patients suffering from it. Regarding the diagnostics, the key expectation is the possibility of predicting occurrence of vasovagal syndrome without the prolonged tilt test. In order to achieve this aim it is crucial to find parameters that allow to predict HUTT results based on the autonomic nervous system response to tilt^50^.

The tilt table test is not a painful test for the patients, but as it is a lengthy procedure it is uncomfortable and inconvenient. Therefore numerous investigations dealing with prediction of the final result of HUTT rely solely on the analysis of the basic signals such as RRI, sBP, dBP in the supine position or a short-time response to tilt^51–56^. For this reason our research is focused on identification of parameters which could successfully predict the final result of HUTT based on the response to tilt^50^. Mallat *et al*. proposed a criterion of a negative tilt test result based on the heart rate (HR) analysis in first 6 minutes following an upright tilt. According to their conclusions the slight increase of HR (≤18 bpm) in the first 6 minutes is an indication of negative HUT result. As interesting as it is, unfortunately this is useful only for a limited group of patients who suffer from unexplained episodes of syncope^55^. Other researchers find that detection of oscillations in blood pressure during HUT can indicate the test outcome. The hemodynamic instability was observed in passive HUT and ntg provocation in patients who experience syncope in test with isopropenol. No blood pressure oscillations were observed in patients with negative result of HUT with isopropanol provocation^53^. HUT test results are most commonly studied on the basis of HRV analysis. However the results are often inconclusive. Erfemof *et al*. showed the difference between patients with positive HUT outcome in passive test and test with ntg provocation. They observed decrement of parasympathetic activity in patients with positive response on ntg-provocation. Such reaction did not occur in patients with negative response on that test^57^. Many researches did not find significant differences in HRV parameters when comparing patients with positive and negative HUT outcome and patients with different types of VVS^52,58–60^. In some studies investigators showed higher sympathetic activity in patients with VVS as compared to healthy subject^51,61^ others, however, observed higher parasympathetic activity^51,52,61^. The described investigations indicate that the analysis of HUTT outcome prediction should focus on more than a single biosignal. Separate analyses of ECG and blood pressure are not effective and have a limited predictive value. Vitrag *et al*. combined analyses of RRI and sBP^51,62^. They proposed an algorithm that would predict VVS during a tilt test. It is based on a simultaneous analysis of trends of RRI and sBP and their low-frequency power LF^62^. That algorithm give an indication of an oncoming syncope about 2 minutes before its occurrence. This research refers to very important field of syncope investigation. The possibility of syncope prediction even shortly before its occurrence would allow to improve the quality of patient’s life. A special device using such algorithm could alert the patient who would then self-administer the medication and lie down^51,62^. Our investigations were based on the analysis of four signals recorded simultaneously during the test. We combined the traditional statistics of the signals with nonlinear analysis. The ideal parameter in terms of syncope diagnosis should enable early identification of patients who will develop syncope based on measurements made in supine position.

In the first stage of our analysis we compared average values of RRI, sBP, dBP and SV in the indicated phases of HUTT. Our findings confirm the results previously reported by other researchers^16,50^. We observed a significant decrease in RRI and SV (Fig. 2A(a,c) (RRI, SV: p < 0.0001) in the HUTT(+) group just after the tilt (phase IIa), accompanied by an increase in sBP and dBP (Fig. 2A(b,d) (sBP: p = 0.0003, dBP: p < 0.0001). Comparing phase III against phase I, we found significantly lower values of RRI (p < 0.0001), sBP (p = 0.039) and SV (p < 0.0001) in the former. Also, the values of blood pressure (sBP, dBP) and stroke volume (SV: p = 0.018) were significantly higher in phase IIa, compared with phase III (Fig. 2A) (sBP: p < 0.0001, dBP: p < 0.0001). In the pre-syncopal phase HUTT(+) patients displayed no significant changes in RRI (p = 0.33).

A slightly different pattern of changes was however observed in patients who presented syncope following with nitroglycerin provocation. The HUTT(−) group presented a statistically significant decrease in SV (p = 0.004) with a concomitant increase in blood pressure (sBP (p < 0.0001), dBP (p < 0.0001) in response to tilt (phase I vs phase IIa, Fig. 2B). There were not significant changes in RRI. In the pre-syncopal phase (phase IIb vs phase III) a statistically significant sudden decrease in sBP and dBP occurred also in this group (Fig. 2B HUTT(−): sBP (p = 0.011), dBP (p = 0.015). The changes of RRI and SV were not significant. This group presented significantly lower values of RRI (p < 0.0001) and SV (p < 0.0001) in phase IIa, compared with phase III. In our opinion the decrease in sBP and dBP is the most characteristic predictor of approaching syncope in both groups.

We also ran a comparison of the HUTT(+) vs. HUTT(−) group in the corresponding phases of the test (I, IIa, III). We found statistically significant differences between both groups regarding the pre-syncopal phase III for all analyzed parameters (RRI (p < 0.0001), sBP (p < 0.0001), dBP (p < 0.0001), SV (p = 0.01) (Fig. 5). Patients from the HUTT(−) group had significantly lower values of RRI and SV and higher values of sBP and dBP than patients in the HUTT(+) group, most likely due to the administration of nitroglycerin, because NTG given in a upright position causes further significant reduction of venous return and increase of the heart rhythm, which leads to a vasovagal reaction. Stroke volume was the only parameter that significantly different groups in the phase I (p = 0.01). Significantly higher values of SV were found in the supine position (phase I) in HUTT(−) patients than in their HUTT(+) counterparts (Fig. 5). This observation suggests impedance measurement as a base for creation of markers of syncope occurrence in the tilt test, possibly to be detected as early as in the first stage of the test (in the supine position). The vasovagal reaction mechanism is different depending on whether NTG is used or not despite the absence of differences in RRI and BP. Spontaneous syncope (positive passive HUTT result) and NTG induced syncope are considered as equivalent for diagnostic purposes. The described above difference of SV values in supine position and differentiated pattern of changes of SV during the HUT test show the difference between two groups of patients which in medical classifications have the same diagnostic conclusion (syncope during HUTT).

Our main investigations were focused on the assessment of the parameters’ irregularity in successive HUTT phases in HUTT(+) vs. HUTT(−) patients, using Sample Entropy as a measure of irregularity. The entropy is related to the complexity of the data. The value of entropy depends on the type of analyzed data: for random, uniformly scattered data it is the largest, for random series of different probability distributions it is smaller and finally it drops to the value about 0 for highly predictable systems.

Firstly we computed Sample Entropy for the data (RRI, sBP, dBP and SV) and shuffled data (RRIsh, sBPsh, dBPsh and SVsh) in each phase and for each group. The results of the calculations are presented in Table 1. The entropies calculated for the real data differ depending on the phase and the group. In HUTT(+) group the mean value of SampEn (RRIsh) ranges: from 1.5 ± 0.4 in phase III to 2.1 ± 0.2 in phase I. For the remaining parameters the values are between 1.8 and 2.1. In HUTT(−) the mean value of SampEn for shuffled data is not lower than 1.8 and not greater than 2.1. In each phase we also compared the entropies for real and shuffled data, the results are presented in the Fig. 3. In both groups in each phase SampEn for real data was significantly lower than the entropy of shuffled data. Such results indicate that the entropy for the real data is related to the complexity of the analyzed hemodynamic parameters and not only represents their variability.

Figure 4(A,B) presents the mean values of SampEn (RRI), SampEn (sBP), SampEn (dBP) and SampEn (SV) for the HUTT(+) (Fig. 4A) and HUTT(−) (Fig. 4B) group. There was a significant decrease in SampEn (RRI) in successive phases of HUTT in both groups. In the HUTT(+) group there was a significant decrease in SampEn (RRI) and SampEn (SV) in response to tilt (phase I vs phase IIa, Fig. 4A) (p < 0.0001). In HUTT(−) patients, however, we found a significant drop only in SampEn (RRI) (p < 0.011) in response to tilt (Fig. 4B). Comparing phase IIb against phase III in HUTT(−) group, we did not find significant differences in SampEn values of measured signals. When comparing phases IIa and III, HUTT(+) patients displayed significant changes in all parameters. We observed lower values of SampEn (RRI), SampEn (sBP) and SampEn (dBP) but significantly higher SampEn (SV). We assume that the substantial decrease in entropy occurring for RRI, sBP and dBP, just before syncope results from the decreasing irregularity of these parameters, which in turn leads to undesirable stability of the system, typical for syncope. However there are different patterns of SampEn changes in the analyzed groups. The most interesting changes, however, concerned the oscillating dynamics of SampEn (SV) in both groups. The highest degree of irregularity occurred in phase I (SampEn over 1). Then it transiently decreased to 0.8 in response to tilt (Table 1), only to increase again in the subsequent phases and eventually decrease in the pre-syncope phase, with mean entropy values slightly over 1, ranging from 0.8 to 1.16 (Table 1). The dynamic changes displayed by SampEn (SV) in contrast to other parameters, shifted our attention to the role of the impedance measurement in the diagnosis of syncope^63–65^.

Figure 5 presents comparisons of SampEn (RRI), SampEn (sBP), SampEn (dBP) and SampEn (SV) between the HUTT(+) and HUTT(−) groups in successive HUTT phases. Despite the significant differences observed between the successive phases within each group separately, we did not find many statistically significant differences between the groups. Thus, it can be concluded that changes in the parameters’ irregularity are quite similar in each group. The only significant differences were noted for SampEn (sBP) (p = 0.0008) and SampEn (dBP) (p = 0.001), with their values significantly higher in the HUTT(−) vs. HUTT(+) group (SampEn (sBP): 0.51 vs 0.8; SampEn(dBP): 0.4 vs 0.6, Table 1).

We also focus on changes in the dynamics of entropy in all phases of the test. We analyzed SampEn of different parameters in each phase of HUTT (I, IIa, III) using a 100-beat sliding window advancing by 1 beat on a beat-to-beat basis. In Fig. 6 one can find graphic plots of entropy values (SampEn (RRI), SampEn (sBP), SampEn (dBP) and SampEn (SV) presented separately for each HUTT phase and comparing both study groups with each other (Fig. 6a–c).

A brief analysis shows that the values of SampEn calculated in the sliding windows are higher than that calculated for each 250-beat phase. The observed differences are strictly connected with the SampEn sensitivity to data length *N*. According to the numerical experiments the clear stabilization of the entropy is achieved for 2000 points, but the recommended data length is over 200. The data length less than 75–100 is not recommended for SampEn calculation, therefore we used 100-beat sliding windows and our attention was rather focused on the changes of the entropy in each phase, than the analysis of its values^66^.

The character of the changes of entropy in both study groups in phases I and IIa was very similar. The plots of the entropies overlap and have similar values in the whole phase. The highest value of entropy noted in the HUTT(+) group (around 1.6) was observed for RRI in phase I (Fig. 6a, Table 1). In phase IIa SampEn (RRI) decreased to a value of approximately 1 and in the pre-syncope phase even lower, down to 0.3. The changes in SampEn (RRI) in the HUTT(−) group were quite similar, with the only difference of the decrease in SampEn (RRI) down to 0.4, occurring just after nitroglycerine intake (phase IIb) and remaining at this level until syncope occurrence (Fig. 6b). The entropy of systolic blood pressure (SampEn (sBP) and diastolic blood pressure (SampEn (dBP) in phase I and IIa in both study groups was similar and it remained below 1. Similar changes in the regularity of SV (SampEn (SV) in phase I and IIa were noted for both groups (Fig. 6a,b), however the character of the changes in phase IIa is of particular interest and is different from other parameters. In response to tilt SampEn (SV) rapidly increased from 0.55 to 1.55 and remained at this level thereafter. Occurrence of syncope was directly preceded by a slow decrease in the analyzed entropies (Fig. 6c) in HUTT. The changes in SampEn (RRI), SampEn (sBP) and SampEn (dBP) assessed for the HUTT(+) group were similar, but they significantly differed from those found in the HUTT(−) group. In HUTT(−) group, both SampEn (sBP) and SampEn (dBP) were much higher than SampEn (RRI) and it seems that in the case of the HUTT(−) group the irregularity of blood pressure is on a higher level than the irregularity of heart rhythm. In the HUTT(+) group syncope occurred at much lower values of SampEn (sBP) and SampEn (dBP) as compared with the HUTT(−) group. In this pre-syncope phase the entropy of each parameter decreased noticeably in the HUTT(+) group (Fig. 6c). In this group the vagal activity of autonomous nervous system modulates the irregularity of heart rhythm much more than in the HUTT(−) group, where we observe a fairly slight decrease of SampEn (RRI), SampEn(sBP) and SampEn(dBP). Moreover SampEn (SV) slightly increases at the beginning, but then it stagnates at the level of 1.4 until the onset of syncope. Stroke volume (SV) is an important parameter which distinguishes between vasovagal reactions with application of NTG and without. Both the SV value before the tilt test and the dynamics of sample entropy of the SV during HUTT suggest that the mechanism of these reactions is different.

Simultaneous measurement of ECG and blood pressure are “standard measurements” during HUTT due to necessity of VASIS classification. The idea of application of hemodynamics parameters based on ICG measurement in prediction and interpretation HUT test outcome was proposed by other researches^10,67–72^. Bellard *et al*. found significant differences between patients with positive and negative HUTT outcome in ventricular ejection in response to tilt^71^. But they did not find significant differences in response to tilt in parameters related to SV. Shen *et al*. found decrease in SV and increase in heart rate (HR) in patients with positive HUTT outcome with provocation. Patients with negative HUTT outcome had non-significant HR increase^73^. On the contrary, Novak *et al*.^50^ observed a decrease of SV in patients with and without positive HUTT outcome. The significant decrease of SV was also observed by Zadi *et al*. after 2 minutes and 5 minutes of tilt^74^. Koźluk *et al*. also found in the 5th minute of the tilt significant decrease in hemodynamic parameters in patients with positive HUTT outcome^50^. The analysis mentioned above described HUTT in two groups of patients: with and without positive outcome. The positive HUTT group consisted of the patients with a positive result of the passive test and the test with provocation as opposed to patients with negative HUTT results. The presented results are not consistent and lead to a conclusion that the role of parameters based on ICG measurement in HUTT test is still not sufficiently explained and requires further investigations.

In our investigation we tried to find a difference between passive HUTT and HUTT with provocation. There was searching of much more subtle differences limited to patients suffer from syncope. Therefore the observed differences in SV and Sample Entropy of blood pressure seem to have great potential in finding a key to prediction of HUTT outcome.

Our investigations are an example of a practical application of Sample Entropy for vasovagal syncope assessment. We can conclude that entropy and its changes calculated for the heart rhythm and blood pressure are not able to identify patients that fall or do not fall in the passive tilt table test. Essential differences were only observed for stroke volume. Therefore, only this parameter (or another one obtained from ICG measurement) appears to be a promising candidate for future investigation. It can be beneficial to analyze other measures of complexity for ICG measurements.

## Limitations

The analysis presented in this paper skips the issue of gender. The uneven share in patient gender mirrors the gender-related differences in syncope occurrence seen in the general population. We are also conscious of the influence of the age on the hemodynamic parameters and their complexity. In such cases, we would need to divide the study population into smaller subgroups to include gender and age in our investigation. Such division, however, would not allow us to reach statistical significance.

## References

[1] Raj SR, Freeman R (2010). Highlights in clinical autonomic neurosciences:faiting and fainters-who, why to do when they show up in the emergency departament?. Autonomic Neuroscience.

[2] Brignole M (2018). ESC Guidelines for the diagnosis and management of syncope. Eur. Heart J..

[3] Parry SW (2009). The Newcastle Protocols 2008: An update on head-up tilt table testing and the management of vasovagal syncope and related disorders. Heart..

[4] Ganzeboom KS, Colman N, Reitsma JB, Shen WK, Wieling W (2003). Prevalence and triggers of syncope in medical students. Am J Cardiol..

[5] Serletis A, Rose S, Sheldon AG, Sheldon RS (2006). Vasovagal syncope in medical students and their first-degree relatives. Eur Heart J..

[6] Folino A, Migliore F, Marinelli A, Ilceto S, Buja G (2010). Age-related hemodynamic changes during vasovagal syncope. Autonomic Neuroscience..

[7] Mosqueda-Garcia R, Furlan R, Tank J, Fernandez-Violante R (2000). The elusive pathophysiology of neurally mediated syncope. Circulation..

[8] Abboud FM (1993). Neurocardiogenic syncope. NEJM..

[9] Flevari PP (2002). Baroreflexes in vasovagal syncope: two types of abnormal response. Pacing Clin. Electrophysiol..

[10] Alboni P, Alboni M, Bertorelle G (2008). The origin of vasovagal syncope: to protect the heart or to escape predation?. Clin. Auton. Res..

[11] https://www.cnsystems.com/products/task-force-monitor.

[12] Verheyden B, Ector H, Aubert AE, Reybrouck T (2008). Tilt training increases the vasoconstrictor reserve in patients with neurally mediated syncope evoked by head-up tilt testing. European Heart Journal..

[13] Schwalm, T. *Modern tilt table testing and non-invasive monitoring*. ABW Wissenschftsverlag GmbH (2007).

[14] Kenny RA, Ingram A, Bayliss J, Sutton R (1986). Head up tilt: a usefull test for investigating unexplained syncope. Lancet.

[15] Forelo, C. *et al*. Head-up tilt testing diagnosing vasovagal syncope: a meta-analysis. *Int J Cardiol*. 2–9 (2012). 10.1016/j.ijcard.2012.09.023 23041006

[16] Graff B (2015). Entropy measures in the assessment of heart rate variability in patients with cardiodepressive vasovagal syncope. Entropy.

[17] Barbieri, R., Scilingo, E.P. & Valenza, G. *Complexity and Nonlinearity in Cardiovascular Signals*. (Springer, 2017).

[18] Makowiec D, Graff B, Struzik Z (2017). Multistructure index in revealing complexity of regulatory mechanism of human cardiovascular system at rest and orthostatic stress in healthy humans. Physica A.

[19] Shin DG (2006). Prediction of paroxysmal atrial fibrillation using nonlinear analysis of the R-R interval.Dynamics before the spontaneous onset of atrial fibrillation. Circ. J..

[20] Piskorski J, Guzik P (2011). The structure of heart rate asymmetry: deceleration and acceleration runs. Physiological Measurement.

[21] Ammirati F, Colivicchi F, Santini M (2000). Diagnosing syncope in clinical practice. Implementation of a simplified diagnostic algorithm in a multicentre prospective trial – the OESIL 2 study. Eur Heart J..

[22] Blanc JJ (2002). Prospective evaluation and outcome of patients admitted for syncope over a 1 year period. Eur Heart J.

[23] Blanc JJ, L’Her C, Gosselin G, Cornily JC, Fatemi M (2005). Prospective evaluation of an educational programme for physicians involved in the management of syncope. Europace.

[24] Brignole M (2006). A new management of syncope: prospective systematic guideline-based evaluation of patients referred urgently to general hospitals. European Heart J..

[25] Crane SD (2002). Risk stratification of patients with syncope in an accident and emergency department. Emerg Med J.

[26] Disertori M (2003). Management of patients with syncope referred urgently to general hospitals. Europace.

[27] Sarasin FP (2001). Prospective evaluation of patients with syncope: a population-based study. Am J Med..

[28] Stephenson, J. *Fits and Faints*, Blackwell Scientific Publications. (Oxford 1990).

[29] Lieshout JJ, Wieling W, Karemakar JM, Eckberg DL (1991). The vasovagal response. Clin Sci..

[30] Oberg B, Thoren P (1872). Increased activity in the aventricular receptors during hemorrhage or occlusion of caval veins in the cat: a possible cause of vasovagal reaction. Acta Physiol Scand..

[31] Clausius, R. The Mechanical theory of heat – with its applications to the steam engine and to physical properties of bodies (1867).

[32] Shannon CE (1948). A mathematical theory of communication. Bell System Technical Journal.

[33] Pincus SM (1991). Approximate entropy as a measure of system complexity. Proc. Natl. Acad. Sci..

[34] Ferrario M, Signorini MG, Magenes G, Cerutti S (2006). Comparison of Entropy-Based Regularity Estimators: Application to the Fetal Heart Rate Signal for the Identification of Fetal Distress. IEEE Trans. Biomed. Eng..

[35] Graff B, Graff G, Kaczkowska A (2012). Entropy measures of heart rate variability for short ECG datasets in patients with congestive heart failure. Acta Phys. Pol. B Proc. Suppl..

[36] Liu C (2011). Comparison of different threshold values r for approximate entropy: application to investigate the heart rate variability between heart failure and healthy control groups. Physiol. Meas..

[37] Sarlabous, L. *et al*. Interpretation of the approximate entropy using fixed tolerance values as a measure of amplitude variations in biomedical signals. *IEEE Conf. Proc. Eng. Med. Bio. Soc*. 5967–5970 (2010). 10.1109/IEMBS.2010.5627570 21096950

[38] Richman JS, Moorman JR (2000). Physiological time-series analysis using approximate entropy and sample entropy. Am.J. Physiol. Heart Circ. Physiol..

[39] Pincus S (1995). Approximate entropy as a complexity measure. Chaos.

[40] Tuzcu V, Nas S, Borklu T, Ugur A (2006). Decrease in the heart rate complexity prior to the onset of atrial fibrillation. Europace.

[41] Cavanaugh JT, Kochi N, Stergiou N (2010). Nonlinear analysis of ambulatory activity patterns in community-dwelling older adults. J. Gerontol. Ser. A Biol. Sci. Med. Sci..

[42] Sosnoff JJ, Goldman MD, Motl RW (2010). Real life walking impairment in multiple sclerosis: preliminary comparison of four methods for processing accelerometry data. Mult. Scler..

[43] Pincus S, Huang W (1992). Approximate entropy-statistical properties and applications. Commun. Stat. Theory Methods..

[44] Fleisher LA, Di Pietro JA, Johnson TR, Pincus S (1997). Complementary and noncoincident increases in heart rate variability and irregularity during fetal development. Clin. Sci..

[45] Fleisher LA, Pincus SM, Rosenbaum SH (1993). Approximate entropy of heart rate as a correlate of postoperative ventricular dysfunction. Anesthesiology.

[46] Buszko K, Piątkowska A, Koźluk E, Opolski G (2017). Entropy in investigation o f vasovagal syndrome in passive head up tilt test. Entropy.

[47] Brignole M (2000). New classification of hemodynamics of vasovagal syncope: beyond the VASIS classification. Analysis of the pre-syncopal phase of the tilt test without and with nitroglycerin challenge. Vasovagal syncope international study. Europace.

[48] Fitzpatrick AP, Theodorakis G, Vardas P, Sutton R (1991). Methodology of head-up tilt testing in patients with unexplained syncope. J.Am Coll Cardiol..

[49] https://www.physionet.org/physiotools/ (accessed on 15 January 2017).

[50] Koźluk E (2014). Early hemodynamic response to the tilt test in patients with syncope. Clin. Res..

[51] Virag N, Sutton R, Vetter R, Markowitz T, Erickson M (2006). Prediction of vasovagal syncope from heart rate and blood pressure trend and variability: experience in 1,155 patients. HeartRythm.

[52] Duplyakov D, Golovina G, Sysuenkova E, Garkina S (2011). Can the result of a tilt test be predicted?. Cardiology Journal.

[53] Hausenloy DJ (2009). Blood pressure oscillations during tilt testing as a predictive marker of vasovagal syncope. Europace.

[54] Gimeno-Blanes, F. J. *et. al*. *EURASIP Journal on Advances in Signal Processing* 2011–33 (2011).

[55] Mallat Z (1997). Prediction of head-up tilt test result by analysis of early heart rate variations. Circulation.

[56] Gimeno-Blanes FJ (2011). Early prediction of tilt test outcome, with support vector machine non linear classifier, using ECG, pressure and impedance signals. Computing in Cardiology.

[57] Efremov Kristian, Brisinda Donatella, Venuti Angela, Iantorno Emilia, Cataldi Claudia, Fioravanti Francesco, Fenici Riccardo (2014). Heart rate variability analysis during head-up tilt test predicts nitroglycerine-induced syncope. Open Heart.

[58] Kouakam C (1999). Inadequate sympathovagal balance in response to orthostatism in patients with unexplained syncope and a positive head up tilt test. Heart.

[59] Mehlsen J, Kaijer MN, Mehlsen AB (2008). Autonomic and electrocardiographicchanges in cardioinhibitory syncope. Europace.

[60] Evrengul H, Tavli HV, Evrengul H, Tavli T, Dursunoglu D (2006). Spectral and time-domain analysis of heart-rate variability during head-upright tilt-table testing in children with neurally mediated syncope. Pediatr Cardiol..

[61] Salameh E (2007). Heart ratevariability and vasovagal syncope. Ann Cardiol Angeiol..

[62] Virag, N. *et al*. Predicting vasovagal syncope from heart rate and blood pressure: a prospective study in 140 subjects. *HeartRythm*. published online April (2018). 10.1016/j.hrthm.2018.04.032 29715516

[63] Cybulski Gerard (2011). Validation of the Ambulatory Impedance Cardiography Method. Ambulatory Impedance Cardiography.

[64] Cybulski G, Strasz A, Niewiadomski W, Gąsiorowska A (2012). Impedance cardiography: recent advancements. Cardiol. J..

[65] Parry SW (2009). Impedance cardiography: a role in vasovagal syncope diagnosis?. Age Ageing.

[66] Yentes JM (2013). The Appropriate use of Approximate Entropy and Sample Entropy with short data sets. Ann. Biomed. Eng..

[67] Novak V, Honos G, Schondorf R (1996). Is the heart “empty’ at syncope?. Auton Nerv Syst..

[68] Liu JE (2000). Left ventricular geometry and function preceding neurally mediated syncope. Circulation.

[69] Yamanouchi Y (1996). Changes in left ventricular volume during head-up tilt in patients with vasovagal syncope: an echocardiographic study. Am Heart J..

[70] Leonelli FM (2000). False positive head-up tilt: hemodynamic and neurohumoral profile. JACC.

[71] Bellard E (2003). Changes in the transthoracic impedance signal predict the outcome of a 70 degrees head-up tilt test. Clin Sci..

[72] Deegan BM (2007). Orthostatic hypotension: a new classification system. Europace.

[73] Shen WK (2000). Distinct hemodynamic profiles in patients with vasovagal syncope: a heterogeneous population. J Am Coll Cardiol..

[74] Zaidi A, Benitez D, Gaydecki PA, Vohra A, Fitzpatrick AP (2000). Haemodynamic effects of increasing angle of head up tilt. Heart.

